# Tamaño de repeticiones CTG no aumentado en la transmisión de un padre con alelo expandido: falsa sospecha de fenómeno de contracción

**DOI:** 10.1515/almed-2022-0120

**Published:** 2023-03-02

**Authors:** Nuria Goñi Ros, Paula Sienes Bailo, Ricardo González Tarancón, Loreto Martorell Sampol, Silvia Izquierdo Álvarez

**Affiliations:** Servicio de Genética y Bioquímica Clinica, Hospital Universitario Miguel Servet, Zaragoza, España; Servicio de Medicina Genética y Molecular, Hospital Sant Joan de Déu, Barcelona, España

**Keywords:** *DMPK*, distrofia miotónica tipo 1, limitaciones de la TP-PCR, repeticiones del trinucleótido

## Abstract

**Objetivos:**

La distrofia miotónica tipo 1, conocida también como enfermedad de Steinert, es un desorden multisistémico crónico, degenerativo e incapacitante de expresividad clínica muy variable provocado por una expansión heredada de manera autosómica dominante de la repetición del triplete citosina-timina-guanina, localizada en la región 3′ no codificante del gen *DMPK* (19q13.3).

**Caso clínico:**

En este estudio, presentamos el caso de una familia con varias expansiones de la repetición CTG intergeneracionales, con un caso adicional de falsa sospecha de fenómeno de contracción, debido a las limitaciones de la técnica TP-PCR.

**Conclusiones:**

La inestabilidad meiótica de las repeticiones de (CTG)_n_ provoca anticipación genética. De este modo, a lo largo de las sucesivas generaciones, se ha hallado un incremento del tamaño de la mutación DM1 y un fenotipo más severo en los individuos afectados. Aunque es extremadamente infrecuente, en la transmisión de padres a hijos, también puede producirse una disminución en el número de repeticiones CTG, siendo esta más frecuente en la transmisión paterna.

## Introduction

La distrofia miotónica tipo 1 (DM1; OMIM#160900), conocida también como enfermedad de Steinert, es un desorden multisistémico crónico, degenerativo e incapacitante de gravedad variable provocado por una expansión heredada de manera autosómica dominante de la repetición del trinucleótido citosina-timina-guanina, localizada en la región 3′ no codificante del gen *DMPK (19q13.3).* El ARN resultante adquiere una ganancia de función tóxica debido a las repeticiones expandidas de CUG, que forman focos ribonucleares, compuestos por estructuras en horquilla que se unen y secuestran las proteínas de unión a ARN [1].

La DM1, también conocida como enfermedad de Steinert, con una prevalencia global de 1 por cada 800 habitantes, es el tipo más común de distrofia muscular de inicio en la edad adulta. Los pacientes con DM1 suelen experimentar debilidad y desgaste esquelético, hiperexcitabilidad (miotonía), cataratas, deterioro cognitivo, problemas gastrointestinales, complicaciones cardiovasculares y resistencia a la insulina, entre otros síntomas [2]. Dado que el espectro fenotípico es amplio y los síntomas son variables, la identificación y validación de indicadores de resultado adecuados para la investigación clínica es todo un reto [3]. Mientras que, en sujetos sanos, las repeticiones de CTG varían entre 5 y 37, en los pacientes con enfermedad de Steinert, este triplete de CTG se repite entre 50 y más de 3000 veces. Un alelo con una longitud de repeticiones de 38–50 CTG se considera un alelo en rango de premutación, que indica una mayor inestabilidad hacia mayores repeticiones expandidas patológicamente. La inestabilidad meiótica de las repeticiones de (CTG)_n_ produce anticipación genética, lo que resulta en un mayor tamaño de la mutación DM1 y un fenotipo más grave en los sujetos afectados en las sucesivas generaciones [4]. Por lo general, cuanto mayor sea el número de repeticiones, mayor será la gravedad de la enfermedad. Aun cuando no existe un paralelismo entre estos dos factores, la correlación genotipo-fenotipo es mayor a partir de las 400 repeticiones CTG. Entre las diversas clasificaciones diagnósticas se encuentran la DM1 congénita, infantil/de la infancia, juvenil, con debut en la edad adulta, y en la edad adulta tardía/asintomática, cada una de ellas determinadas según criterios de edad al inicio de la enfermedad y gravedad de los síntomas [5, 6]. Aunque raramente, se puede producir una disminución en el tamaño de las repeticiones CTG durante la transmisión de padres a hijos, normalmente en la transmisión por vía paterna [7]. La TP-PCR es una alternativa efectiva a las limitaciones de otros métodos diagnósticos, como la hibridación Southern y la PCR convencional, ya que estos métodos requieren de un gran volumen de muestra, mayores concentraciones de ADN, poseen una mayor complejidad y, en consecuencia, mayores tiempos de respuesta. Así mismo, la PCR convencional no distingue entre homocigotos y heterocigotos, y ninguno de los dos detecta claramente mosaicismos con alelos de diferentes tamaños. La TP-PCR permite realizar una cuantificación exacta de los alelos en un rango de entre 5 y 150 repeticiones de CTG. La PCR clásica en gel de agarosa puede cuantificar hasta 100 repeticiones de CTG, aunque la precisión en el número de repeticiones es menor que con la TP-PCR. Aunque, con la hibridación Southern, el error suele ser mayor en los alelos dentro de ese rango, para tamaños de expansión >150 CTG, resulta la mejor opción, ya que permite una cuantificación exacta del número de repeticiones. De este modo, la principal limitación de la TP-PCR es que no permite cuantificar el número de repeticiones de los alelos expandidos (en el caso de la enfermedad de Steinert, alelos con un tamaño superior a las 150 repeticiones).

En el presente estudio, presentamos el caso de una familia con varias expansiones intergeneracionales de la repetición CTG, con el hallazgo adicional de un niño con una falsa sospecha de contracción del alelo de DM1, debido a las limitaciones de la técnica de TP-PCR.

## Caso clínoco

**Figura 1:**
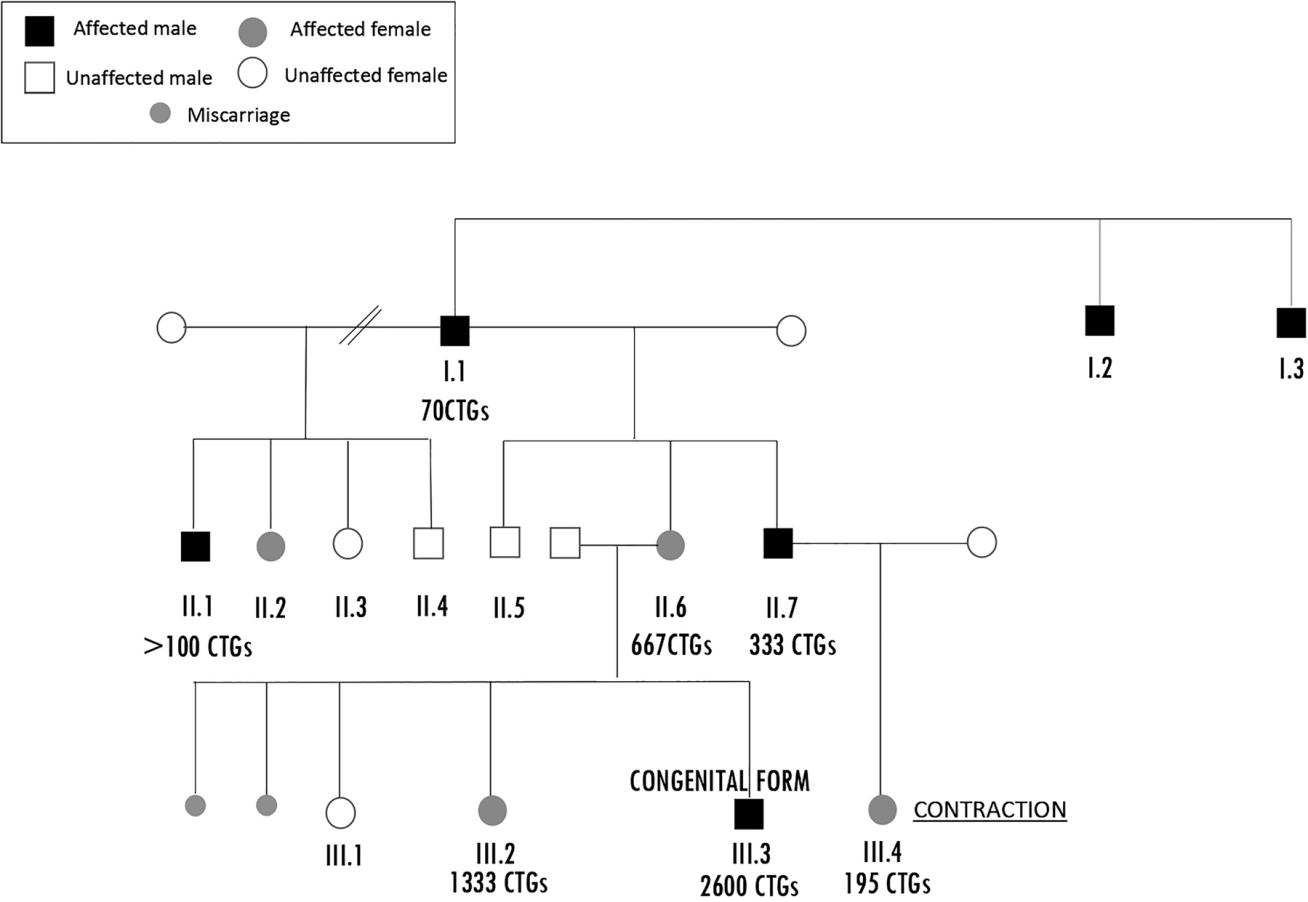
Árbol genealógico detallando los familiares afectos de Steinert y el número de interrupciones CTG.

El caso índice (Figura 1. II.6) es el de una mujer de 33 años, embarazada de 23 semanas, con facies miopática, fenómeno miotónico en las manos, astenia/fatiga y labio superior en forma de V invertida. Como antecedentes familiares de interés, su padre y abuelo padecían cataratas y tenía un hermano con síntomas similares. Debido a la ausencia de relación con la rama paterna de la familia, no se pudieron obtener más datos de interés. La paciente había tenido dos abortos en el momento de la consulta, y una de sus hijas tenía retraso en el desarrollo e hipotonía. Ante la sospecha de enfermedad de Steinert, los neurólogos solicitaron un estudio genético para confirmar o descartar la presencia de expansiones del gen *DMPK*, para establecer o no el diagnóstico de DM1. Para ello, en nuestro laboratorio se emplean las técnicas de PCR (Adellgene Myotonic Dystrophy Screening kit, Diagnóstica Longwood) y la PCR de tripletes cebados, TP-PCR (Adellgene^®^ Myotonic Dystrophy Confirmatory kit, Diagnóstica Longwood). El análisis de fragmentos se realizó en un secuenciador ABI 3130xl empleando el programa GeneMapper 4.0. El límite de detección del tamaño del alelo mediante TP-PCR fue de 150 CTG, y la precisión en la estimación del tamaño mediante PCR fue de ± 1 para el rango normal de tamaño del alelo. En este caso, el informe de TP-PCR reflejaba un resultado compatible con el diagnóstico de enfermedad de Steinert, ya que en la muestra de la paciente se encontró un alelo de 5 ± 1 repeticiones CTG y un segundo alelo expandido con más de 150 repeticiones CTG (unas 667 CTG, confirmadas mediante hibridación de Southern).

En ese momento, se decidió realizar un estudio genético de las hijas de nuestro caso índice, para analizar la presencia de alelos asociados a la DM1. En una de ellas (Figura 1. III.1), se hallaron dos alelos dentro del rango normal (6 ± 1 CTG, y 13 ± 1 CTG), lo que descartaba la enfermedad. La otra hija (Figura 1. III.2) era una niña de 5 años con retraso psicomotor e hipotonía, cuyo estudio reveló un alelo con más de 150 repeticiones CTG confirmadas mediante Southern, cuantificándose 1333 repeticiones CTG. Dado que la mujer estaba embarazada en el momento del diagnóstico, se llevó a cabo también un estudio prenatal, lo que permitió detectar una forma congénita de la enfermedad (Figura 1. III.3), con un alelo con 2600 repeticiones CTG. A pesar de ello, la pareja decidió seguir adelante con el embarazo. Su hijo nació con hipotonía, acidosis respiratoria, dificultades respiratorias e hiperbilirrubinemia. Con 15 días de vida, precisó nutrición parenteral, así como una traqueotomía combinada con una gastrostomía. Finalmente, el niño falleció a los cuatro meses de edad, debido a bradicardia e hipoperfusión.

**Figura 2:**
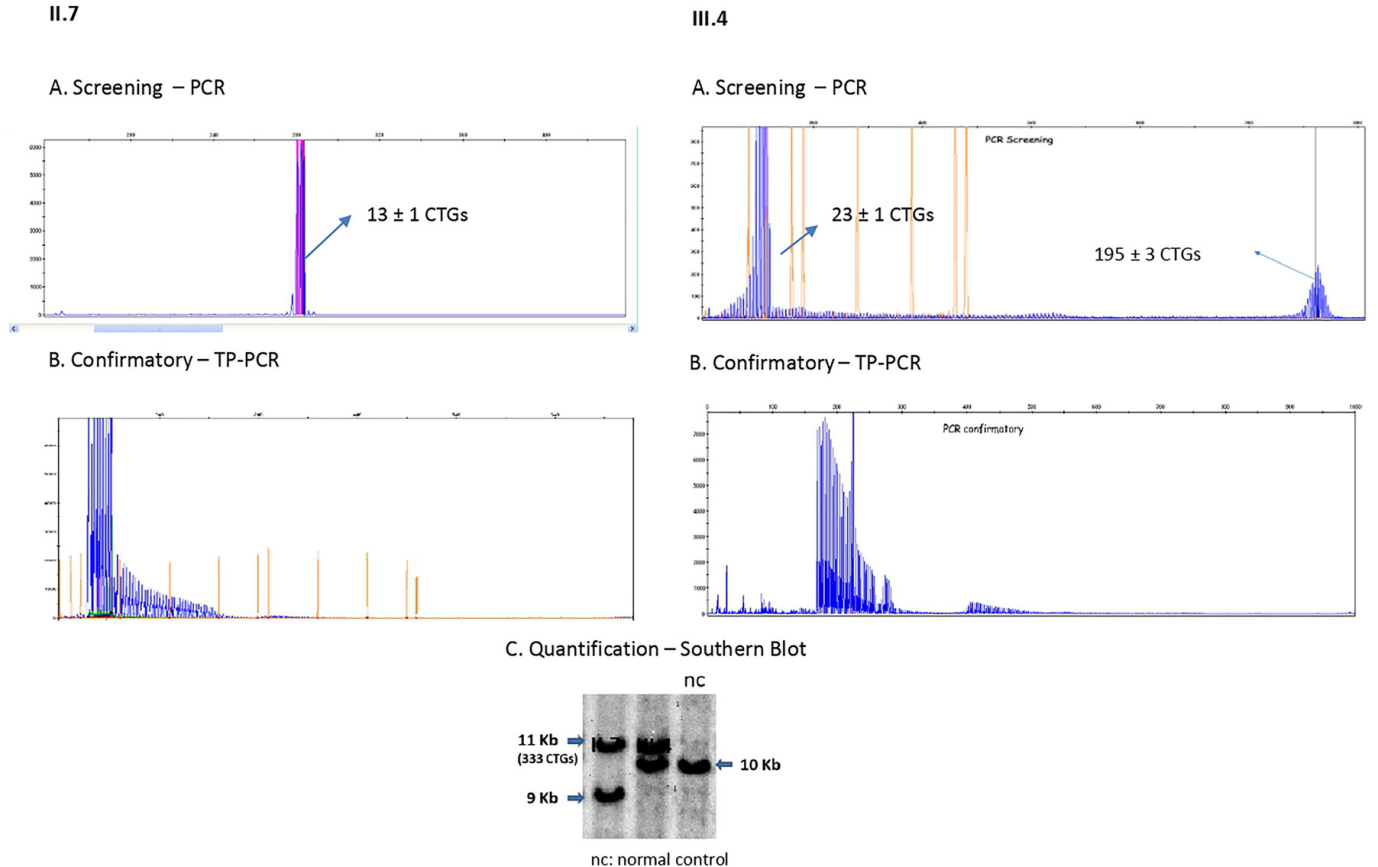
Resultados de las pruebas genéticas en los sujetos II.7 y III.4 (cribado mediante PCR, confirmación mediante TP-PCR y cuantificación mediante Southern).

Para realizar un seguimiento de este caso de DM1 congénito, se realizó un estudio genético al tío del niño (hermano de nuestro caso índice) (Figura 1. II.7). Se trataba de un varón de 29 años con fenómeno miotónico en la cara y alopecia frontal. Como resultado, encontramos un alelo en rango normal y otro expandido (333 CTG). El paciente y su esposa estaban esperando un bebé, por lo que continuamos con el estudio de la familia, con el propósito de averiguar si el bebé también era portador de la expansión del trinucleótido CTG en el gen *DMPK*. Para tal fin, se realizó un test prenatal, que reveló un alelo normal y otro con unas 195 repeticiones de CTG (Figura 1. III.4), no siendo posible realizar un análisis de Southern para cuantificar el alelo expandido, debido a que la muestra prenatal de ADN resultó insuficiente. A la luz de estos resultados, y siendo preciso descartar un posible mosaicismo de la línea germinal, antes de considerar seriamente un fenómeno de contracción del número de copias, se realizó una prueba de Southern tras el nacimiento. La cuantificación del alelo expandido reveló 333 repeticiones de CTG (al igual que su padre, pero sin haberse producido la expansión del alelo paterno) (Figura 2B), lo que nos llevó a descartar tanto la posibilidad de un mosaicismo como un fenómeno de contracción del número de repeticiones de CTG.

## Discusión

La DM1 se hereda de manera autosómica dominante, por lo que el riesgo de que la progenie de un paciente afectado herede la mutación es del 50%. Siendo la repetición expandida de CTG en el rango anormal en el locus de la DM1 una de las secuencias más inestables en el genoma humano, presentando con frecuencia tasas de mutación en la longitud de la línea germinal superiores al 95%, esta tiende a aumentar de tamaño en las sucesivas generaciones. Por esta razón, los descendientes pueden heredar longitudes de repeticiones considerablemente mayores que las presentes en el progenitor transmisor. Este fenómeno es conocido como anticipación genética, donde la gravedad de la enfermedad aumenta y/o la edad de inicio se reduce de una generación a otra [8, 9]. Las repeticiones de CTG también son significativamente inestables en el soma, y las mutaciones parecen acumularse a través de múltiples cambios de longitud [7]. De este modo, parece razonable suponer que la inestabilidad somática condicionada por la expansión y la edad, limitada a unos tejidos concretos, explica la especificidad de los tejidos afectados y la naturaleza degenerativa de los síntomas [9]. Clínicamente, la anticipación es un fenómeno sorprendentemente recurrente en un gran número de familias con DM1. En el presente estudio, presentamos el caso de una familia con varias expansiones intergeneracionales de las repeticiones de CTG, en la que, una vez uno de sus miembros recibió el diagnóstico de DM1, la estrecha vigilancia médica y familiar llevó al diagnóstico temprano de la enfermedad en los descendientes, permitiendo un mejor manejo de los pacientes y un asesoramiento genético adecuado para la planificación familiar.

Es sabido que la DM1 congénita se suele producir por la transmisión por vía materna de la mutación (especialmente, a partir de los alelos expandidos de >300 repeticiones CTG), por lo general acompañada de un extraordinario aumento intergeneracional en el tamaño de las repeticiones de CTG. Este caso familiar, en el que se observa una forma congénita con transmisión materna de la expansión de las repeticiones CTG lo demuestra, respaldando también la teoría de la implicación de algún factor materno en la patogénesis de la DM1.

Sin embargo, en las familias con DM1, el tamaño de las repeticiones CTG no siempre aumenta en las sucesivas generaciones, no habiéndose realizado estudios sistemáticos de las características de estos casos. Con una prevalencia de 4,2–6,4% en la población, estimada en un solo estudio poblacional a lo largo de 20 años, también se ha descrito una disminución en el tamaño de las repeticiones de CTG durante la transmisión de padres a hijos, principalmente en la transmisión por vía paterna (10%). Informamos de un caso de transmisión paterna, donde se sospechó un fenómeno de contracción en la hija de un sujeto afectado. Dicha sospecha inicial surgió tras realizar una prueba prenatal, donde la TP-PCR reveló un máximo de 195 repeticiones de CTG. Posteriormente, dicha sospecha se descartó al realizar la prueba de Southern tras el nacimiento. Este caso demuestra la importancia de la prueba de Southern a la hora de confirmar el diagnóstico de DM1, ya que, aunque la TP-PCR es más económica y más habitual en la mayoría de los laboratorios, esta determina si la longitud del alelo está en rango normal o patológico, pero no permite cuantificar con precisión el número de repeticiones CTG, cuando estas se encuentran por encima de 150 o 200 [10]. La poca frecuencia con la que se produce el fenómeno de contracción (nunca se ha detectado en nuestro laboratorio, tras más de 550 estudios genéticos de DM1), nos lleva a señalar la importancia de descartar falsas identificaciones, debido tanto a mosaicismos germinales como a expansiones superiores a las 150 repeticiones CTG, que la mayoría de los kits de TP-PCR no detectan.

En la DM1, la longitud de la expansión de las repeticiones predice la gravedad de las manifestaciones clínicas y la edad a la que la enfermedad debuta. Volviendo a nuestro caso, aunque el alelo paterno expandido no había sufrido un fenómeno de contracción en los descendientes, resulta muy curioso el hecho de que el padre y la hija presentaran exactamente el mismo número de repeticiones de CTG.

En conclusión, este caso evidencia las limitaciones de las técnicas de cribado (PCR cuantitativa o TP-PCR) en el procedimiento diagnóstico de la DM1, evidenciando el papel fundamental de los métodos de confirmación (método Southern) y la importancia del sexo del progenitor afectado, que determina el tamaño de las repeticiones de CTG en la descendencia. La gravedad de la DM1 viene determinada fundamentalmente por la longitud del alelo del progenitor, representando esta última el 64% de la variación en la edad al inicio, existiendo una sencilla relación lineal [9]. Sin embargo, podría existir un factor materno desestabilizante, que permite a las repeticiones CTG seguir expandiéndose, aumentando así la probabilidad de transmisión de la DM 1 en la progenie, pudiendo existir además un factor paterno que restringe la expansión y previene la aparición de la DM1. Sin duda, son precisos más estudios para alcanzar una mayor comprensión de la dinámica mutacional asociada a los loci de las repeticiones de secuencias expandidas, incluyendo la identificación de otros modificadores genéticos implicados. Los resultados de dichos estudios, así como una mayor comprensión de la relación genotipo-fenotipo en la DM1, así como los trastornos de inestabilidad microsatelital, permitirán la clasificación genética, la evaluación pronóstica y el manejo clínico de estos pacientes y sus familias.
